# First Trimester Urine and Serum Metabolomics for Prediction of Preeclampsia and Gestational Hypertension: A Prospective Screening Study

**DOI:** 10.3390/ijms160921520

**Published:** 2015-09-08

**Authors:** Marie Austdal, Line H. Tangerås, Ragnhild B. Skråstad, Kjell Å. Salvesen, Rigmor Austgulen, Ann-Charlotte Iversen, Tone F. Bathen

**Affiliations:** 1 Department of Circulation and Medical Imaging, Faculty of Medicine, Norwegian University of Science and Technology, 7491 Trondheim, Norway; E-Mail: marie.austdal@ntnu.no; 2 St. Olavs Hospital, Trondheim University Hospital, 7006 Trondheim, Norway; E-Mail: line.tangeras@ntnu.no; 3 Centre of Molecular Inflammation Research, Department of Cancer Research and Molecular Medicine, Norwegian University of Science and Technology, 7491 Trondheim, Norway; E-Mails: rigmor.austgulen@ntnu.no (R.A.); ann-charlotte.iversen@ntnu.no (A.-C.I.); 4 Department of Laboratory Medicine Children’s and Women’s Health, Faculty of Medicine, Norwegian University of Science and Technology, 7491 Trondheim, Norway; E-Mail: ragnhild.skrastad@ntnu.no; 5 National Center for Fetal Medicine, Department of Obstetrics and Gynecology, St. Olavs Hospital, Trondheim University Hospital, 7030 Trondheim, Norway; E-Mail: pepe.salvesen@ntnu.no; 6 Department of Obstetrics and Gynecology, Clinical Sciences, Lund University, 221 00 Lund, Sweden

**Keywords:** NMR spectroscopy, preeclampsia, metabolomics, PLS-DA, gestational hypertension, prediction, first-trimester screening, biomarker

## Abstract

Hypertensive disorders of pregnancy, including preeclampsia, are major contributors to maternal morbidity. The goal of this study was to evaluate the potential of metabolomics to predict preeclampsia and gestational hypertension from urine and serum samples in early pregnancy, and elucidate the metabolic changes related to the diseases. Metabolic profiles were obtained by nuclear magnetic resonance spectroscopy of serum and urine samples from 599 women at medium to high risk of preeclampsia (nulliparous or previous preeclampsia/gestational hypertension). Preeclampsia developed in 26 (4.3%) and gestational hypertension in 21 (3.5%) women. Multivariate analyses of the metabolic profiles were performed to establish prediction models for the hypertensive disorders individually and combined. Urinary metabolomic profiles predicted preeclampsia and gestational hypertension at 51.3% and 40% sensitivity, respectively, at 10% false positive rate, with hippurate as the most important metabolite for the prediction. Serum metabolomic profiles predicted preeclampsia and gestational hypertension at 15% and 33% sensitivity, respectively, with increased lipid levels and an atherogenic lipid profile as most important for the prediction. Combining maternal characteristics with the urinary hippurate/creatinine level improved the prediction rates of preeclampsia in a logistic regression model. The study indicates a potential future role of clinical importance for metabolomic analysis of urine in prediction of preeclampsia.

## 1. Introduction

Hypertensive disorders of pregnancy, including preeclampsia and gestational hypertension, are major causes of maternal morbidity and mortality, and affect up to 10% of pregnant women [1,2,3]. Early identification of women at high risk of preeclampsia might enable potential prophylactic treatment to reduce or avoid the onset of symptoms [4,5]. Late onset preeclampsia (occurring after 34 weeks of pregnancy) is more common, and has lower detection rate [6]. Predictive models for late onset preeclampsia have employed a combination of maternal characteristics, biochemical and biophysical markers at 11^+0^–13^+6^ weeks of gestation, to predict the syndrome at 30%–60% sensitivity [5,6,7,8]. Skråstad *et al.* [9] have previously found a combination of mean arterial pressure (MAP), maternal age and uterine artery pulsatility index (UtAPI) to be 38.5% predictive of preeclampsia in a cohort of women at gestational weeks 11^+0^–13^+6^. New and improved predictive biomarkers are warranted. Gestational hypertension is often included in the disorder spectrum of preeclampsia, particularly if other symptoms are present. Clinical findings in gestational hypertension are often intermediate between normal pregnancy and preeclampsia [1]. In general, placental, renal, or hepatic involvement are not present in gestational hypertension, and outcomes are better for mother and baby [1].

Metabolomics represents a “top-down” view of the metabolism, which more closely characterises the phenotype of the organism than genomic and proteomic applications. Metabolomics is the detection and semi-quantitation of low molecular weight metabolites present in cells, tissues or body fluids, using high throughput analysis platforms such as proton nuclear magnetic resonance (^1^H NMR) spectroscopy or Mass Spectrometry (MS) [10,11]. Recent interest has mounted in the metabolomics approach to predict and characterize preeclampsia. Early and late preeclampsia has been predicted using serum from weeks 11^+0^–13^+6^ of pregnancy in combination with maternal markers [12,13], and markers of preeclampsia have been found in urine and serum in the second trimester using metabolomics [14,15,16,17]. To date, no studies have attempted to predict hypertensive disorders in pregnancy using ^1^H NMR analysis of urine and serum from early pregnancy in a complete prospective cohort of women.

The aim of this study was to evaluate whether metabolic profiles of urine and serum collected from a cohort of women at gestational week 11^+0^–13^+6^ could predict preeclampsia and/or gestational hypertension. Secondly, we aimed to elucidate the metabolic changes that may accompany the early stages of these hypertensive disorders of pregnancy.

## 2. Results

### 2.1. Characteristics of the Study Participants

A total of 640 women (585 nulliparous and 55 parous women) attended the study visit between 11^+0^and 13^+6^ weeks gestation. A flow chart describing the women included in the analysis is shown in Supplementary Figure S1. After exclusions for conditions appearing at or after the study visit as described in [9], and technical reasons (failed acquisitions or missing samples), 599 women remained in total with 587 urine samples and 591 serum samples. One excluded urine sample was from a woman who developed gestational hypertension.

Characteristics of the study participants for each pregnancy outcome group are shown in Table 1. Twenty-six women (4.3%) later developed preeclampsia and 21 women (3.5%) later developed gestational hypertension. Of the nulliparous women, 3.8% developed preeclampsia and 2.9% gestational hypertension. Of the multiparous women, 12.2% developed preeclampsia and 9.8% gestational hypertension. One woman experienced early onset preeclampsia (delivery <34 weeks gestation) and one woman with gestational hypertension delivered before 34 weeks gestation. Two of the preeclamptic women had neonates classified as small for gestational age. Body mass index (BMI) at study enrolment was higher in women later developing gestational hypertension, and gestational age at birth and birth weights were lower for the neonates born in preeclamptic pregnancies. MAP was higher in women who later developed preeclampsia or gestational hypertension, but below the definition of chronic hypertension.

**Table 1:** Characteristics of the study participants at time of enrolment related to pregnancy outcome.

Characteristics (Stratified Per Pregnancy Outcome)	Preeclampsia	Gestational Hypertension	Normotensive Pregnancies	*p*-value ^a^
Number of women included	26	21	552	-
Age in years, median (IQR)	26 (7)	28 (6)	28 (5)	NS
BMI at enrolment in kg/m^2^, median (IQR)	24.8 (5.6)	27.1 (7.6)	23.5 (4.9)	<0.01
Smoking, *n* (%) ^b^	4 (15.3)	2 (9.5)	63 (11.4)	NS
MAP at enrolment, median (IQR)	87.0 (11.1)	92.1 (8.1)	82.7 (8.8)	<0.001
UtAPI at enrolment, median (IQR)	1.75 (0.70)	1.49 (0.39)	1.46 (0.52)	<0.05
GA at enrolment, weeks, median (IQR)	13.1 (0.6)	13.1 (0.7)	12.9 (0.9)	NS
GA at delivery, weeks, median (IQR)	38.0 (3.1)	40.1 (1.3)	40.2 (1.9)	<0.001
Birth weight, g, median (IQR)	3243 (705)	3460 (1096)	3475 (600)	<0.01

Abbreviations: BMI, body mass index; GA, gestational age; IQR, interquartile range; MAP, mean arterial pressure; NS, not significant; UtAPI, uterine artery pulsatility index. ^a^ *p*-values calculated by nonparametric Kruskal-Wallis test for continuous variables or Fishers exact test for categorical variables; ^b^ The women were asked at the study visit whether they had smoked at any time during the pregnancy.

**Figure 1:**
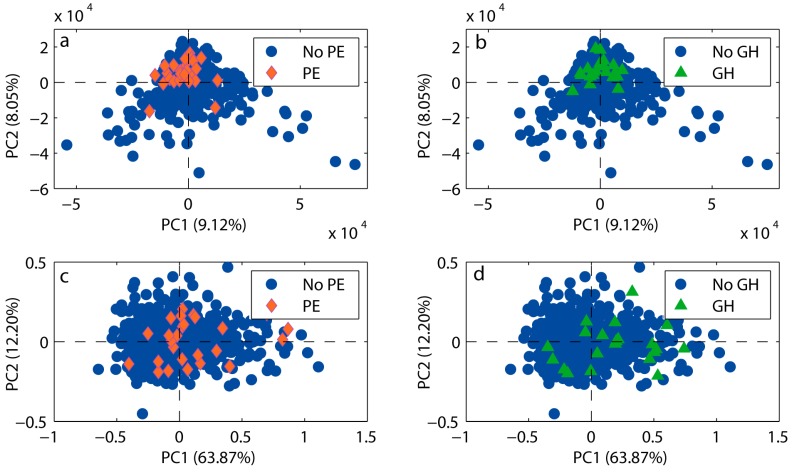
Score plots from principal component analysis (PCA) of urine and serum spectra. PCA score plots of the first and second principal components (PCs) with the percent variance explained, performed on urine samples (**a**,**b**) and serum samples (**c**,**d**). Urine samples gave a clustering of women set to develop preeclampsia (PE) (red diamonds, *n* = 26) or gestational hypertension (GH) (green triangles, *n* = 21), while serum samples gave no apparent clustering. Samples from women without hypertensive disorders of pregnancy are shown in blue circles *n* = 552.

Identification of 54 urine metabolites and 30 serum metabolites was achieved (Supplementary Tables S1 and S2). The exploratory principal component analysis (PCA) (Figure 1) of the urine and serum ^1^H NMR spectra showed a characteristic clustering of urine samples from women who later developed preeclampsia or gestational hypertension, indicating a difference in urinary metabolic profiles between healthy and later hypertensive pregnancies. No apparent clustering was seen in serum samples.

### 2.2. Metabolic Biomarkers in Urine

**Figure 2:**
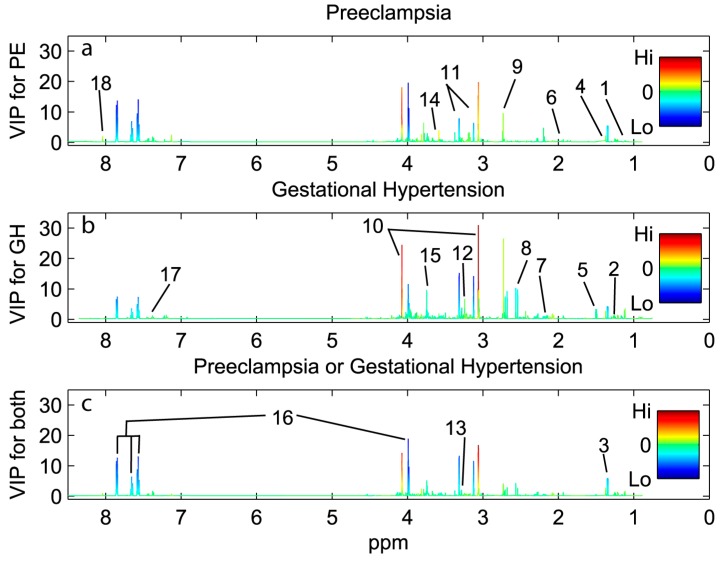
Urine ^1^H NMR variables involved in predicting preeclampsia (PE), gestational hypertension (GH) and both combined using partial least squares discriminant analysis (PLS-DA). The variable importance in projection (VIP) scores for each variable (part per million, ppm) are shown on the vertical axis, with higher VIP scores meaning increasing importance in the predictive model. The variables are colored by the loadings from the corresponding PLS-DA model. Red means increasing levels of metabolite in the indicated condition and blue meaning decreasing levels. Metabolites by number: 1, 4-deoxyerythronic acid; 2, 4-deoxythreonic acid; 3, lactate and threonine; 4, α-hydroxyisobutyrate; 5, alanine; 6, acetate and lysine; 7, glutamine; 8, citrate; 9, dimethylamine; 10, creatinine; 11, proline betaine; 12, carnitine (tentative); 13, betaine; 14, glycine 15, ascorbic acid; 16, hippurate; 17, phenylacetylglutamine; 18, histidine.

Preeclampsia, gestational hypertension and both combined were predicted with urine metabolic profiles using partial least squares discriminant analysis (PLS-DA). The prediction parameters are given in Table 2, and additional model characteristics (latent variables, permutation results) are given in Supplementary Table S3). At 10% false positive rates (FPR) using metabolomics analyses with variable selection, preeclampsia could be predicted at 51% sensitivity from first trimester urine samples, gestational hypertension with 40% sensitivity, and both combined at 37% sensitivity. The loading plots from PLS-DA pinpoint the metabolites that are different between the modelled groups (Figure 2, Table 2). Scores and loading plots for the PLS-DA models are shown in Supplementary Figures S2–S4. Women that later developed preeclampsia had increased urine levels of creatinine, glycine, 4-deoxythreonic acid, α-hydroxyisobutyrate, histidine and dimethylamine and decreased hippurate, lactate and proline betaine. For women developing gestational hypertension there was an additional decrease of urinary citrate excretion.

**Table 2:** Urine metabolite multivariate models predicting preeclampsia and/or gestational hypertension ^a^.

Hypertensive Pregnancy Outcome	Accuracy (%)	Specificity (%)	Sensitivity (%)	Sensitivity at 10% FPR (%)	*p*-Value ^b^	Indicated Metabolites ^c^
Preeclampsia (*n* = 26) *vs.* no preeclampsia (*n* = 561)						
Full urine spectra	61.4	65.3	57.5	11.3	<0.01	↑ Crn, Gly, α-HIB, Hist, DMA ↓ Hipp, Lac/Thr, ProlB
VIP ≥ 1 variables	68.2	60.1	76.3	23.8	<0.01	↑ Crn, Gly, α-HIB, Hist, DMA ↓ Hipp, Lac/Thr
CARS variables	70.8	74.2	67.5	51.3	<0.01	↑ Gly, 4-DEA, DMA ↓ Hipp, Lac, Cre, ProlB
Gestational hypertension (*n* = 20) ^d^ *vs.* no gestational hypertension (*n* = 567)						
Full urine spectra	59.1	68.2	50.0	11.7	<0.01	↑ Crn, α-HIB, DMA ↓ Hipp, Lac/Thr, ProlB, Citrate
VIP ≥ 1 variables	63.7	65.7	61.7	16.7	0.01	↑ Crn, α-HIB, DMA ↓ Hipp, Lac/Thr, ProlB, Citrate
CARS variables	63.8	89.3	38.3	40.0	0.04	↑ DMA ↓ PAG, Ala
Preeclampsia or gestational hypertension (*n* = 46) *vs.* normotensive (*n* = 541)						
Full urine spectra	61.5	56.1	66.8	14.4	<0.01	↑ Crn, α-HIB, DMA, ↓ Hipp, Lac/Thr, ProlB ↑ Crn, α-HIB, DMA ↓ Hipp, Lac/Thr, ProlB
VIP ≥ 1 variables	64.0	56.2	71.9	20.0	<0.01	
CARS variables	66.4	75.9	56.9	36.9	<0.01	↑ α-HIB, DMA ↓ Hipp, PAG, Lys, Ala

Abbreviations: 4-DEA, 4-deoxythreonic acid; α-HIB, α-hydroxyisobutyrate; Ala, alanine; Cre, creatine; Crn, creatinine; CARS, competitive adapted reweighted sampling; DMA, dimethylamine; FPR, false positive rate; Gly, glycine; Hipp, hippurate; Hist, histidine; Lac, lactate; Leu, leucine; PAG, phenylacetylglutamine; ProlB, proline betaine; Thr, threonine; VIP, variable importance in projection. ^a^ VIP or CARS variable selection was performed, and results were evaluated using accuracy, specificity and sensitivity from double cross validation; ^b^ Model validity was estimated by 100 permutation tests; ^c^ The metabolites are listed as increased (↑) or decreased (↓) in the hypertensive disease pregnancies compared to the pregnancies without the disease; ^d^ One urine sample was missing from the gestational hypertension cases.

### 2.3. Metabolic Biomarkers in Serum

**Figure 3:**
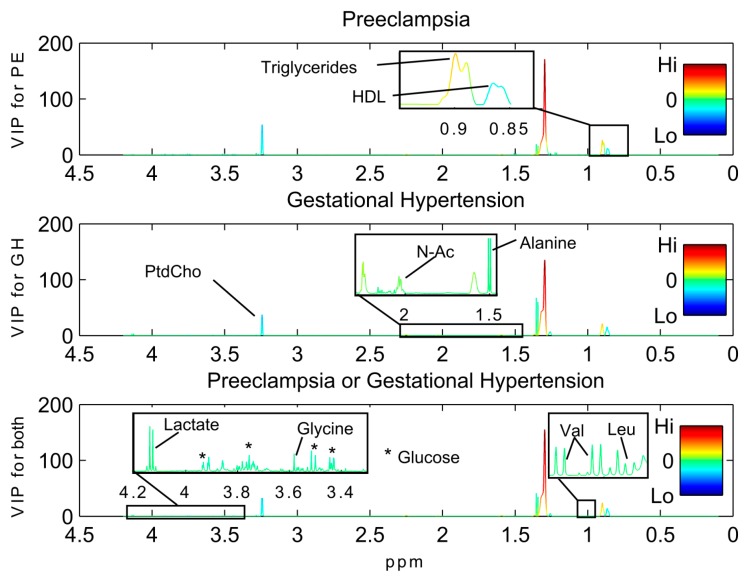
Serum variables involved in predicting preeclampsia and/or gestational hypertension by partial least squares discriminant analysis PLS-DA. The variable importance in projection (VIP) scores for each variable are shown along the vertical axis. The variables are colored by the loadings as described in Figure 3. Abbreviations: HDL, high density lipoprotein; Leu, leucine; N-Ac, N-acetylated carbohydrate side chains of glycoproteins; PtdCho, phosphatidylcholine; Py, pyruvate; Val, valine.

Preeclampsia, gestational hypertension and both combined were predicted with serum metabolic profiles using PLS-DA. The prediction results are given in Table 3, and additional model parameters and permutation results are given in Supplementary Table S4). At 10% FPR, 15%, 33% and 30%, respectively, of preeclampsia, gestational hypertension and both combined could be predicted. The loading plots from PLS-DA pinpoint the metabolites that are different between the hypertensive groups (Figure 3, Table 3). Scores and loading plots for the significant PLS-DA models are shown in Supplementary Figures S5 and S6. Mainly, increased lipid levels were evident in both hypertensive groups, and primarily the increased signals originated from triglycerides. Decreased levels of phosphatidylcholines, with signals originating from lipids in high density lipoproteins (HDL), glucose, lactate and alanine, were also important for the prediction of hypertensive disorders.

**Table 3:** Serum metabolite multivariate models predicting preeclampsia and/or gestational hypertension ^a^.

Hypertensive Pregnancy Outcome	Accuracy (%)	Specificity (%)	Sensitivity (%)	Sensitivity at 10% FPR (%)	*p*-Value ^b^	Indicated Metabolites ^c^
Preeclampsia (*n* = 26), *vs.* no preeclampsia (*n* = 565)						
Full serum spectra	59.4	73.8	45.0	20.0	>0.05	NS
VIP ≥ 1 variables	58.3	70.3	46.3	26.3	>0.05	NS
CARS variables	64.6	65.4	63.8	15.0	0.05	↑ Signals from triglycerides, 3-HB, ↓ Pyruvate, PtdCho, Lac
Gestational Hypertension (*n* = 21) *vs.* no gestational hypertension (*n* = 570)						
Full serum spectra	59.1	74.8	43.3	25.0	>0.05	NS
VIP ≥ 1 variables	58.1	75.0	41.3	22.5	>0.05	NS
CARS variables	66.1	55.0	76.9	33.3	0.02	↑ Signals from triglycerides, ↓ Variables corresponding to HDL, Lac, N-Ac, PtdCho, Glc
Preeclampsia or gestational hypertension (*n* = 47) *vs.* normotensive (*n* = 544)						
Full serum spectra	62.6	70.8	54.4	24.4	0.01	↑ Lipid signals, signals from triglycerides, ↓ Signals from HDL, Glc, Val, Leu, Lac, Ala, PtdCho
VIP ≥ 1 variables	63.0	70.4	55.6	27.5	<0.00	↑ Lipid signals, signals from triglycerides, ↓ Signals from HDL, Glc, Leu, Val, Ala, Lac, PtdCho
CARS variables	64.5	69.1	60.0	30.0	0.02	↑ Variables corresponding to triglycerides, ↓ Lac, PtdCho

Abbreviations: 3-HB, 3-hydroxybutyrate; Ala, alanine; CARS, competitive adaptive reweighted sampling; FPR, false positive rate; GH, gestational hypertension; Glc, glucose; HDL, high density lipoprotein; Lac, lactate; Leu, leucine; N-Ac, N-acetyl glycoproteins; NS, not significant PE, preeclampsia; PtdCho, phosphatidylcholine; Thr, threonine; Val, valine; VIP, variable importance in projection. ^a^ VIP or CARS variable selection was performed, and results were evaluated using accuracy, specificity and sensitivity from double cross validation; ^b^ Model validity was estimated by 100 permutation tests; ^c^ The metabolites are listed as increased (↑) or decreased (↓) in the hypertensive disease pregnancies compared to the pregnancies without the disease.

### 2.4. Combined Metabolic and Clinical Biomarkers Predict Preeclampsia

**Figure 4:**
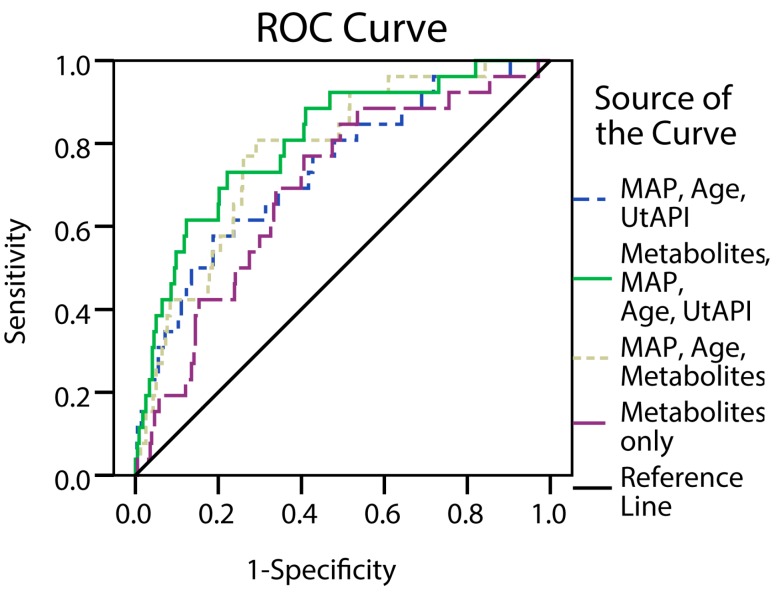
Receiver Operator Characteristic (ROC) curve comparison from logistic regression analyses. Prediction of preeclampsia using logistic regression, with risk of preeclampsia as dependent variable and maternal age and MAP in combination with urinary metabolites (Hippurate and Creatinine) or UtAPI as independent variables. Abbreviations: MAP, Mean Arterial Pressure; UtAPI, Uterine Artery pulsatility index.

Metabolites were combined with maternal characteristics to predict preeclampsia. The best logistic regression models for prediction of preeclampsia are shown in Table 4. Urinary hippurate:creatinine combined with maternal MAP and a variable denoting age >35 or <20 at enrolment gave better prediction rates (AUC 0.778) compared to UtAPI combined with MAP and age (AUC 0.738) (Figure 4). Urine metabolites glycine, proline betaine, lactate, dimethylamine, and 4-deoxythreonic acid to creatinine ratios did not contribute significantly to the logistic regression models.

**Table 4:** Prediction of preeclampsia based on urinary metabolites and maternal characteristics in logistic regression.

Variable	AUC (95% CI)	Sensitivity (%) ^a^	PPV	NPV	*p*-Value ^b^
Hippurate/creatinine ratio ^c^	0.694 (0.595–0.793)	0.192	0.082	0.960	0.004
MAP, age ^d^, UtAPI	0.738 (0.637–0.839)	0.346	0.138	0.967	<0.001
Metabolites, MAP, age ^d^	0.778 (0.695–0.862)	0.423	0.164	0.971	<0.001
Metabolites, MAP, age ^d^, UtAPI	0.807 (0.721–0.893)	0.538	0.200	0.977	<0.001

Abbreviations: AUC, area under the receiver operator characteristic curve; CI, confidence interval; MAP, mean arterial pressure at enrolment; NPV, negative predictive value; PPV, positive predictive value; UtAPI; uterine artery pulsatility index at enrolment. ^a^ Sensitivity is given at 10% false discovery rate; ^b^ Omnibus chi-square significance level of the model; ^c^ Metabolites were chosen based on selection in the multivariate models and the metabolite/creatinine ratios used in logistic regression. The final metabolites selected were hippurate/creatinine ratio in urine; ^d^ Women with maternal age <20 or >35 were categorised as high risk.

## 3. Discussion

Metabolic profiles in urine and serum samples from pregnant women at 11^+0^–13^+6^ weeks gestation were significantly different between women who developed preeclampsia or gestational hypertension, and women with normotensive pregnancies. Both urine and serum metabolic profiles could predict preeclampsia and gestational hypertension, with urine profiles giving the best prediction. Decreased urinary hippurate, increased urinary creatinine, and increased levels of serum lipids were the most important metabolic differences identified in women who later developed preeclampsia or gestational hypertension.

This is the first metabolomics study performed on first trimester urine samples for prediction of preeclampsia and gestational hypertension. Changes in hippurate excretion preceding preeclampsia are novel to this study. Decreased urinary hippurate has been shown to correlate with increased blood pressure [18], and may be related to diet or to blood pressure related changes in the gut microflora, where this metabolite is produced [18,19]. Hippurate and proline betaine excretion to the urine may increase with fruit intake [20], and the reduced excretion of these metabolites in urine of women who later developed preeclampsia may indicate a healthier diet for the normotensive group. The predictive metabolic profile for preeclampsia also included increased glycine and 4-deoxythreonic acid, as well as decreased lactate and creatine. 4-Deoxythreonic acid is a degradation product of 3-hydroxybutyrate [21], which is shown increased in maternal serum in our study. Both preeclampsia and gestational hypertension was associated with an increase in urinary dimethylamine. This metabolite may have dietary origins [22], but is also derived from asymmetric dimethylarginine, a biomarker of increased cardiovascular risk [22,23]. Preeclampsia has been successfully predicted previously with second trimester urine in a NMR metabolomics case-control study [14]. Those results also include increased 4-deoxythreonic acid [14]. However, many of the metabolites were not the same as those highlighted here, including the hippurate to creatinine ratio, which might be attributed to the difference in gestational age between the studies resulting in changing metabolic profiles [24]. Non-metabolomics methods have been used to predict preeclampsia from urine samples, focusing on urinary albumin to creatinine ratios as a measure of kidney function [25], and creatinine levels in urine [26]. Increased urinary creatinine was found to be predictive of preeclampsia in a study as early as 8–10 weeks gestation [26]. Our corresponding findings of increased creatinine in urine may be an effect of the increased BMI and MAP of the women who developed preeclampsia and gestational hypertension [26], or possibly a marker of early renal involvement. Studies have reported increased glomerular filtration rates in pre-hypertensive subjects [27]. Urinary citrate was decreased in women who later developed gestational hypertension. These women had higher BMI, and an inverse relation between citrate excretion and adiposity has recently been found [28]. The metabolic changes preceding preeclampsia and gestational hypertension were otherwise comparable, suggesting that the risk profiles visible to NMR metabolomics are similar for hypertensive disorders of pregnancy, while other measurements such as the UtAPI may to a larger extent reflect a difference between the two diseases.

First-trimester maternal plasma or serum is more commonly used for prediction of preeclampsia by metabolomics methods [12,13,16,29]. In a previous case-control study, a model with maternal characteristics combined with four NMR-measured serum metabolites predicted late onset PE and pointed to disturbed lipid metabolism [12]. MS-based metabolomics studies have found increased serum levels of carnitine, fatty acid and lipid classes to predict preeclampsia [16,29]. However, no previous studies have used metabolomics to predict the related disease gestational hypertension at an early stage of pregnancy. Changes in lipid metabolism evident early in pregnancies of women who develop hypertensive disorders of pregnancy have been established [30,31,32]. Abnormal lipid metabolism may play a role in the aetiology of preeclampsia [32]. Elevated lipid and low-density lipoprotein levels in maternal serum may induce endothelial dysfunction secondary to oxidative stress [30]. Decrease in phosphatidylcholine serum levels related to both gestational hypertension and preeclampsia may indicate altered choline metabolism [33]. Choline is an essential nutrient which functions in phospholipid metabolism, and choline levels may influence inflammation and angiogenesis [33,34,35]. Decreases in phosphatidylcholines have also been found in individuals with increased cardiovascular risk factors [36]. The decrease in serum pyruvate concurrent with the increase in 3-hydroxybutyrate in women who later developed preeclampsia may indicate an early shift in metabolism from glycolysis to ketosis for energy production [37], and this was also reflected in the urine metabolic profiles.

Combining urinary hippurate to creatinine ratios with maternal MAP and age increased the prediction rates of preeclampsia compared to using the uterine artery Doppler measurement with MAP and age. Replacing the UtAPI measurement, which requires skilled ultrasound technicians and time, with an easily accessible urinary marker, would be an advantage especially in low resource areas. However, clinical application of the metabolic profiling prediction method will require confirmation in cohorts from other populations, where the metabolites identified in this study form the basis of prediction. Currently, early identification of women at risk for developing hypertensive pregnancy disorders would enable a closer follow-up of these women [38]. Extensive research is currently examining potential prophylactic treatment, especially for preterm or severe preeclampsia [4,39,40]. In order for these treatments to work, it is important to identify the women at risk for developing the disease at an early time point. This study shows that there is potentially predictive information contained in a simple urine sample.

The prediction of preeclampsia and gestational hypertension using metabolic profiling of urine performed with similar sensitivity as previous approaches using maternal biophysical and biochemical markers on the same cohort [9,41], and in other studies [5,8], while the serum metabolic profiling did not predict the syndromes very well. This may reflect the lower sensitivity of ^1^H NMR spectroscopy to detect small molecular weight metabolites in serum, due to viscosity, lipid signal overlap and lower concentrations of ^1^H NMR-visible metabolites. However, the large contribution to the model from the lipid regions of the spectra indicate that a lipidomic approach could give additional information about the first-trimester maternal serum changes related to preeclampsia.

The major strength of our study was the prospective design with complete follow-up of almost 600 women with medium to high risk of preeclampsia. The multivariate statistical analyses were performed up to standards of the field, with rigid cross validation [42]. Weaknesses of our study include the limited numbers of cases, which is difficult to overcome given the low incidence of the disorders. An advantage with multivariate analyses as used in metabolomics approaches is that the covariance between metabolites is taken into account in the modelling. However, in order to more conveniently translate metabolic findings to the clinic, a selection of metabolites could be made which convey most of the information contained in the source. Combining sets of metabolites with maternal characteristics may improve the prediction rates. Previous publications within the metabolomics field have used case-control studies, or samples obtained at a later gestational age, to demonstrate a difference in metabolic profile before onset of preeclampsia [12,13,16,29,43]. This study aimed to predict two hypertensive disorders of pregnancy in a prospective cohort of women, and this study design more accurately reflects the predictive power of a metabolomics approach than a case-control design. This difference in design may also explain why our results have lower prediction rates compared to previous metabolic case-control studies.

The sensitivity of prediction of preeclampsia using metabolic biomarkers in this study (51% at 10% FPR) was markedly higher than previously published prediction of preeclampsia based on clinical markers such as MAP, maternal age and UtAPI in the same cohort (38.5%) [9] and in another nulliparous cohort (37%) [44]. However, the achieved sensitivity of prediction of preeclampsia shown here is not high enough for clinical implementation, and the presented model warrants validation in other cohorts.

## 4. Experimental Section

### 4.1. Study Population

The study population has previously been described in detail [9,42]. Briefly, pregnant women who were nulliparous or had preeclampsia or gestational hypertension in a previous pregnancy were invited to attend an examination at 11^+0^–13^+6^ weeks of gestation (crown-rump length 45–84 mm). At the study visit participants were interviewed about their health and pregnancy. All participants were weighed, and BMI was calculated in kg/m^2^. The women were asked to fast for one hour before their visit to avoid immediate effects of meals on the metabolic profiles. Venous blood was drawn into non-heparinised tubes, left to clot for 30 min at room temperature, and centrifuged at 1800× *g* for 10 min. A serum sample (0.8 mL) was separated and stored at −80 °C, thawed once and an aliquot of 120 μL was stored at −80 °C. Spot urine samples were collected at the study visit and aliquots (1.8 mL) were stored directly at −80 °C. Blood pressure was measured with a CAS 740 MAX NIBP automated device (CAS Medical systems Inc., Branford, CT, USA) [45]. MAP from the arm with the highest MAP was used. Participants were examined with transabdominal ultrasound with a Siemens ACUSON Antares™ machine (Siemens Medical Solutions Inc., Santa Clara, CA, USA), and the UtAPI was measured [46]. The UtAPI was measured three times on each side, and the average of three measurements on each side was used. The average of the two sides was used in calculations. All scans were carried out by specialized trained midwifes who were certified by the Fetal Medicine Foundation (http://www.fetalmedicine.com). Data on pregnancy outcomes were collected from hospital records. Preeclampsia was defined as systolic blood pressure ≥140 mmHg and/or diastolic blood pressure ≥90 mmHg in combination with proteinuria ≥0.3 g per 24 h measured twice within 4–6 h by dipstick ≥+1, occurring after gestational week 20 [47]. Gestational hypertension was defined as the previously described hypertension occurring without proteinuria after gestational week 20. Early onset preeclampsia was defined as preeclampsia with delivery before 34 weeks of pregnancy, and late onset preeclampsia with delivery after 34 weeks. For estimation of weight deviation at birth, the normal values from Marsal *et al.* were applied [48]. The definition of small for gestational age was birthweight with a mean below two standard deviations (22%). All women gave written informed consent at study entry. The study was approved by the Regional Committee for Medical Research Ethics in mid-Norway, entries REK 2010/102 and 2013/386.

### 4.2. ^1^H NMR Metabolomic Analyses

Laboratory analyses were done after all women had delivered their babies. Urine samples were thawed on ice and centrifuged at 5600× *g* (Sorvall RMC 14; DuPont, Wilmington, NC, USA) for five minutes. The supernatant (540 μL) was mixed with a bacteriostatic buffer (60 μL) (pH 7.4, 1.5 mM KH_2_PO_4_ in D_2_O, 0.1% Trimethyl-Silyl Propionate (TSP), 2 mM NaN_3_) (Receipt from Bruker Biospin AG, Reinstetten, Germany) and transferred to 5 mm NMR tubes (Bruker Biospin, Billerica, MA, USA). Serum samples were thawed on ice. Serum (100 μL) was mixed with a bacteriostatic buffer (100 μL) (pH 7.4 0.075 mM Na_2_HPO_4_, 5 μM NaN_3_, 5 μM TSP) (Bruker Biospin) and transferred to 3 mm NMR tubes.

NMR analysis was performed at the MR Core Facility at the Norwegian University of Science and Technology (NTNU), Trondheim, Norway using a Bruker Avance III Ultrashielded Plus 600 MHz spectrometer (Bruker Biospin GmbH, Rheinstetten, Germany) equipped with a 5 mm QCI Cryoprobe with integrated, cooled preamplifiers for ^1^H, ^2^H and ^13^C. Experiments were fully automated using the SampleJet™ in combination with Icon-NMR on TopSpin 3.1 software (Bruker Biospin). The ^1^H NMR analyses were performed blinded to pregnancy outcomes. ^1^H NMR spectroscopy acquisition parameters are described in Supplementary Table S1. One-dimensional Standard Nuclear Overhauser Effect spectroscopy (NOESY) (noesygppr1d; Bruker Biospin) spectra were acquired on the urine samples for quantitative detection of small molecular weight metabolites. On the serum samples, Carr-Purcell-Meiboom-Gill (CPMG) (cpmgpr1d; Bruker Biospin) spectra were acquired. In CPMG spectra, signals from macromolecules have been filtered out for better detection of small molecular weight metabolites. For additional aid in metabolite identification, two-dimensional spectra were acquired (Supplementary Table S5).

Spectra were automatically Fourier transformed, phased and baseline corrected in TopSpin with a line broadening of 0.3Hz. Spectra were imported to Matlab r2013b (The Mathworks Inc., Natick, MA, USA). Urine spectral regions containing metabolites of interest (δ 0.5 to 9.0 ppm) were extracted and the residual H_2_O and urea signals removed. Peaks in the urine spectra were aligned by the *i*Coshift algorithm [49] with 245 manually chosen intervals, using the spectrum with the highest correlation as the reference [50]. The urine spectra were normalised using probabilistic quotient normalization to account for differences in dilution [51]. Finally the data was pareto scaled and mean centered [52]. Serum spectral regions containing metabolites of interest (δ 0.1 to 4.2 ppm) were extracted and aligned by the left alanine doublet peak at 1.48 ppm. The spectra were normalised to unit area and mean centered. Raw spectral data are available on request.

### 4.3. Statistical Analysis

Statistical analyses were done in Matlab and in Statistical Package for the Social Sciences (SPSS) (version 20.0; SPSS Inc., Chicago, IL, USA). Baseline characteristics of the study population were tested for normality using the Kolmogorov-Smirnov test [53]. Non-normal data was reported as median (25th–75th percentile), normal data as mean (standard deviation), and categorical data as number (percentage) using either the Kruskal-Wallis test, analysis of variance (ANOVA), or Fishers exact test. The metabolic data was explored for clusters and outliers using PCA as described below. The predictive potential of the metabolic profiles for preeclampsia, gestational hypertension, and both combined were evaluated using PLS-DA. The predictive values of urine and serum ^1^H NMR spectra were evaluated by sensitivity at 10% false positive rate and at the best cutoff on the Receiver Operator Characteristic (ROC) curve.

PCA is a powerful method of data extraction which finds combinations of variables, called principal components (PCs), describing the main variation in large data. These are visualised in scores and loading plots, making it possible to visualise high dimensional data using only a few dimensions. The score plots show each spectrum as an object in the PC space, and are useful for identifying clusters, trends and outliers in the dataset. The loading plots show the variable’s contributions for defining each PC. PLS-DA models the relationship between the spectra and class information using multivariate regression methods, and is used to establish prediction models. The metabolites responsible for separation between classes are given by the latent variables (LVs) [54]. Similar to PCA, the resulting model can be visualised in scores and loading plots. Variable Importance in Projection (VIP) is a method of assessing which variables are most important to the prediction. Variables with VIP scores ≤1 can be considered irrelevant to the prediction and excluded [55]. Competitive Adaptive Reweighted Sampling (CARS) is a variable selection method that iteratively selects variable subsets that perform best in cross validated regression [56].

Multivariate models were constructed using PLS Toolbox 7.3.1 (Eigenvector Research, Wenatchee, WA, USA). Preeclampsia, gestational hypertension and both disorders combined were predicted within the entire cohort; e.g., for prediction of preeclampsia the gestational hypertension remained in the control group. The input to the classification models was either full, preprocessed spectra, or sets of variables selected by different variable selection algorithms (VIP or CARS). VIP selection was performed by creating a five-fold cross validated model, then selecting variables with VIP >1 to use in a further model [55]. CARS variable selection was performed with five-fold cross validation and 50 Monte Carlo samplings [57]. The classification models were evaluated using a double cross validation procedure [57]. A set of samples (20%) is set aside for independent validation of the model (the outer loop). The remaining samples are split into an inner calibration and test set used for determining the optimal number of PLS components as defined by the lowest prediction error. The inner and outer loops are both repeated 20 times. The data was split so that the ratio of case to control samples was the same in validation, calibration and test sets. Mean sensitivity, specificity and classification accuracy were calculated from the outer validation set. The PLS-DA classification results were validated using permutation testing on the classification error rates, with *p*-values ≤0.05 considered significant. Permutation testing performs classification on reshuffled classes, and tests whether those prediction results are significantly different from prediction of randomised classes [57]. One hundred permutations were built for each predictive model.

Finally, urine metabolites found to be important to the multivariate predictions based on high VIP scores and/or CARS selection (hippurate, lactate, dimethylamine, and 4-deoxythreonic acid to creatinine ratios) were quantified by measuring the area under the curve in the ^1^H NMR spectra. The metabolites were input to logistic regression models alone and in combination with maternal characteristics. The maternal variables found to give the best prediction of preeclampsia in the same cohort by Skråstad *et al.* [9] (UtAPI, MAP and maternal age) were combined with selected metabolite ratios, in order to improve the prediction rates for preeclampsia in the cohort. Preeclampsia was predicted using maternal MAP and age as variables in combination with metabolite ratios. Women with maternal age above 35 or less than 20 years were categorised as high risk [58]. The results were compared to prediction rates obtained using UtAPI, MAP and age. Logistic regression analyses were done in SPSS (version 20.0; SPSS Inc., Chicago, IL, USA).

## 5. Conclusions

Metabolic profiling of urine and serum in early pregnancy revealed specific, significant changes in the metabolism of women who later developed preeclampsia or gestational hypertension. Preeclampsia and gestational hypertension could be successfully predicted in early pregnancy using urine and serum metabolic profiles. The consistent changes in the urinary metabolome represent an attractive avenue for clinical prediction, since spot urine is easily accessible. Combining a panel of urine metabolites with maternal characteristics may improve the accuracy of prediction of preeclampsia.

## Supplementary Materials

Supplementary materials can be found at http://www.mdpi.com/1422-0067/16/09/21520/s1.
