# Supplementary material for: First Trimester Urine and Serum Metabolomics for Prediction of Preeclampsia and Gestational Hypertension: A Prospective Screening Study
Source: Int J Mol Sci. 2015 Sep 8;16(9):21520–38. doi: 10.3390/ijms160921520 (PMC4613265; doi:10.3390/ijms160921520)
Supplement: Supplementary file 1 [file ijms-16-21520-s001.pdf]

Supplementary Information

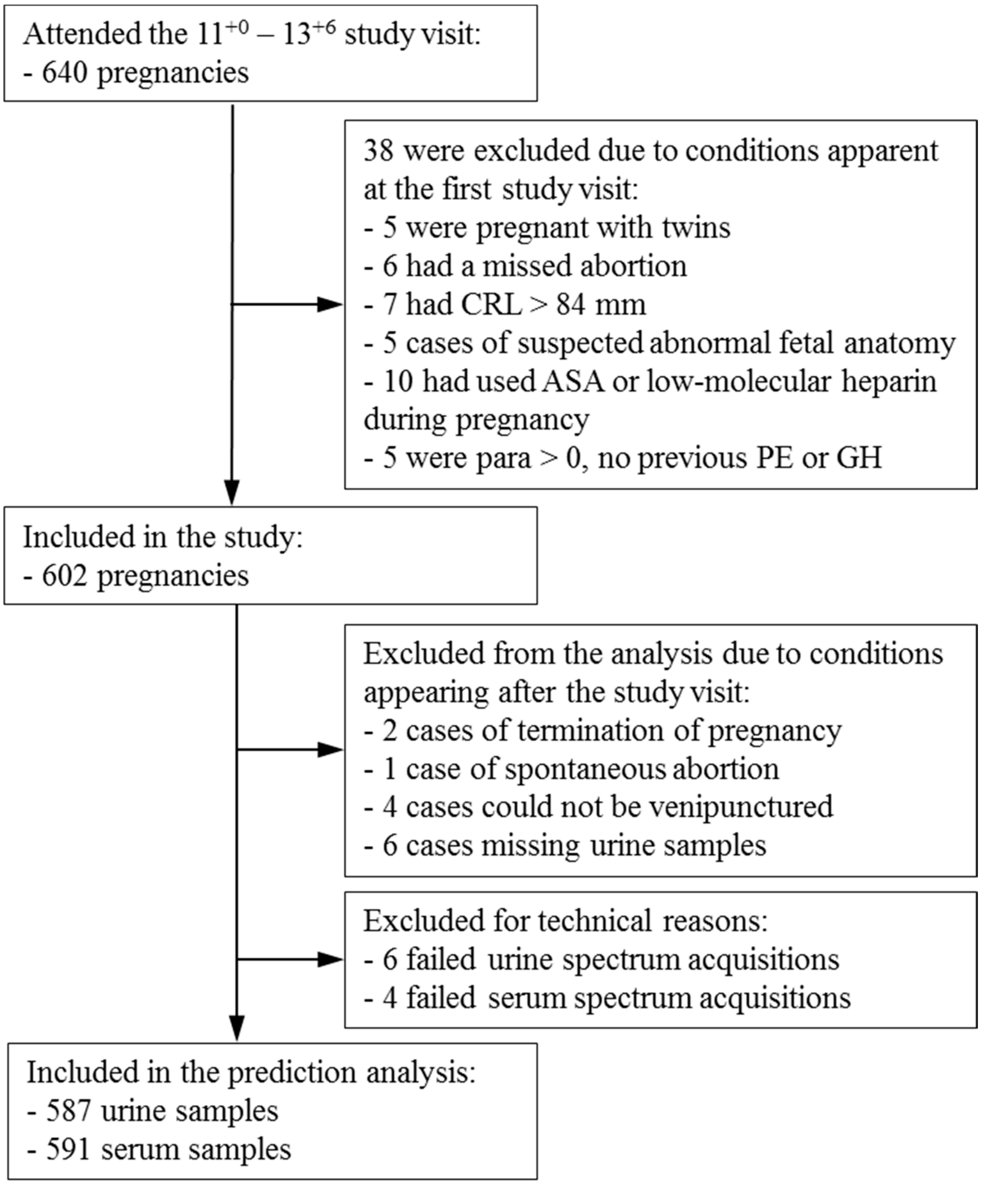

Figure S1. Flow chart describing participants included in the study. Abbreviations: ASA, Acetyl Salisylic acid; CRL, Crown rump length; GH, gestational hypertension; PE, preeclampsia.

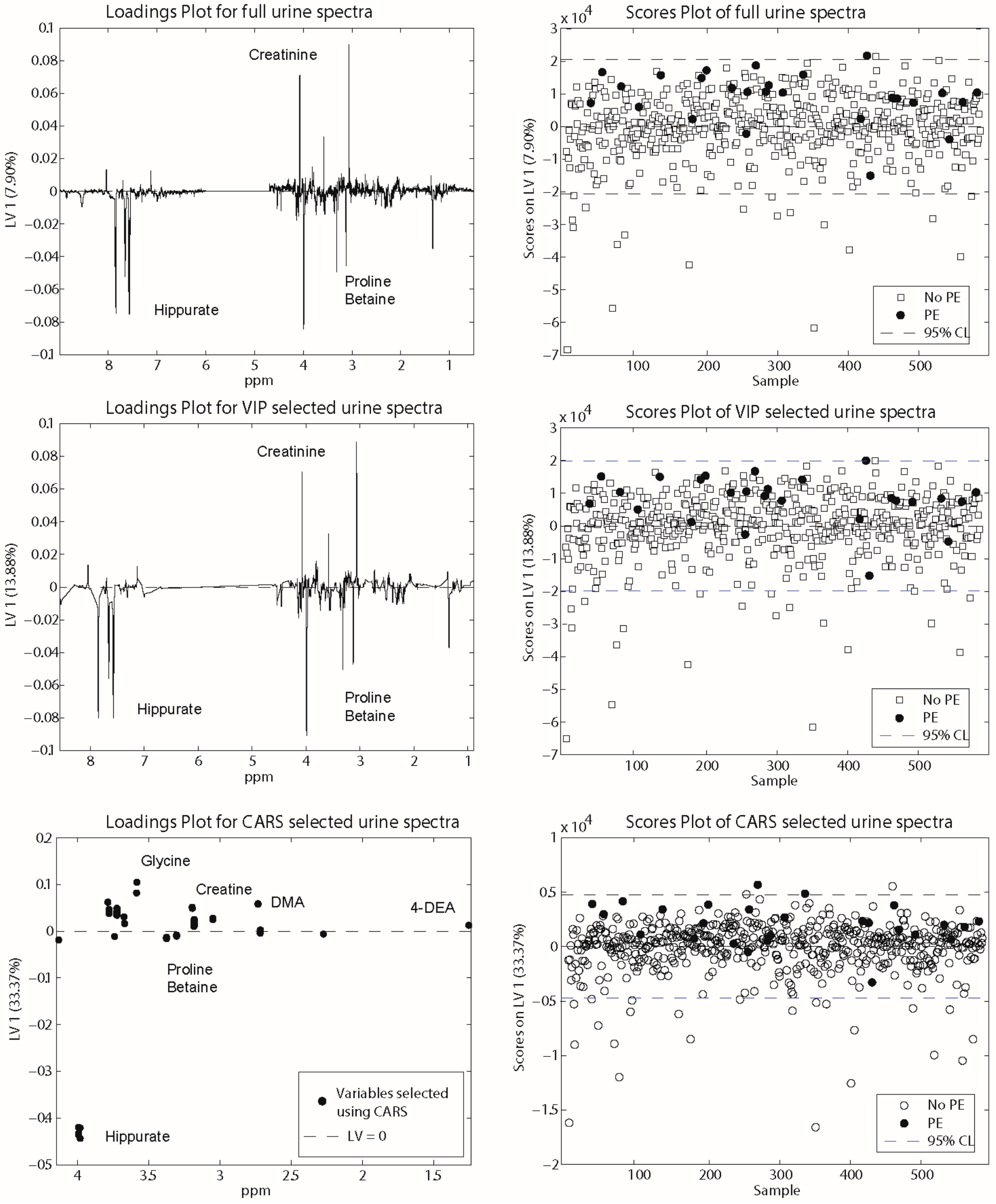

Figure S2. Scores and loadings for models predicting preeclampsia. Abbreviations: 4-DEA, 4-deoxythreonic acid; CARS, competitive adaptive reweighted sampling; CL, confidence limit; DMA, dimethylamine; LV, latent variable; PE, preeclampsia; ppm, parts per million; VIP, variable importance in projection.

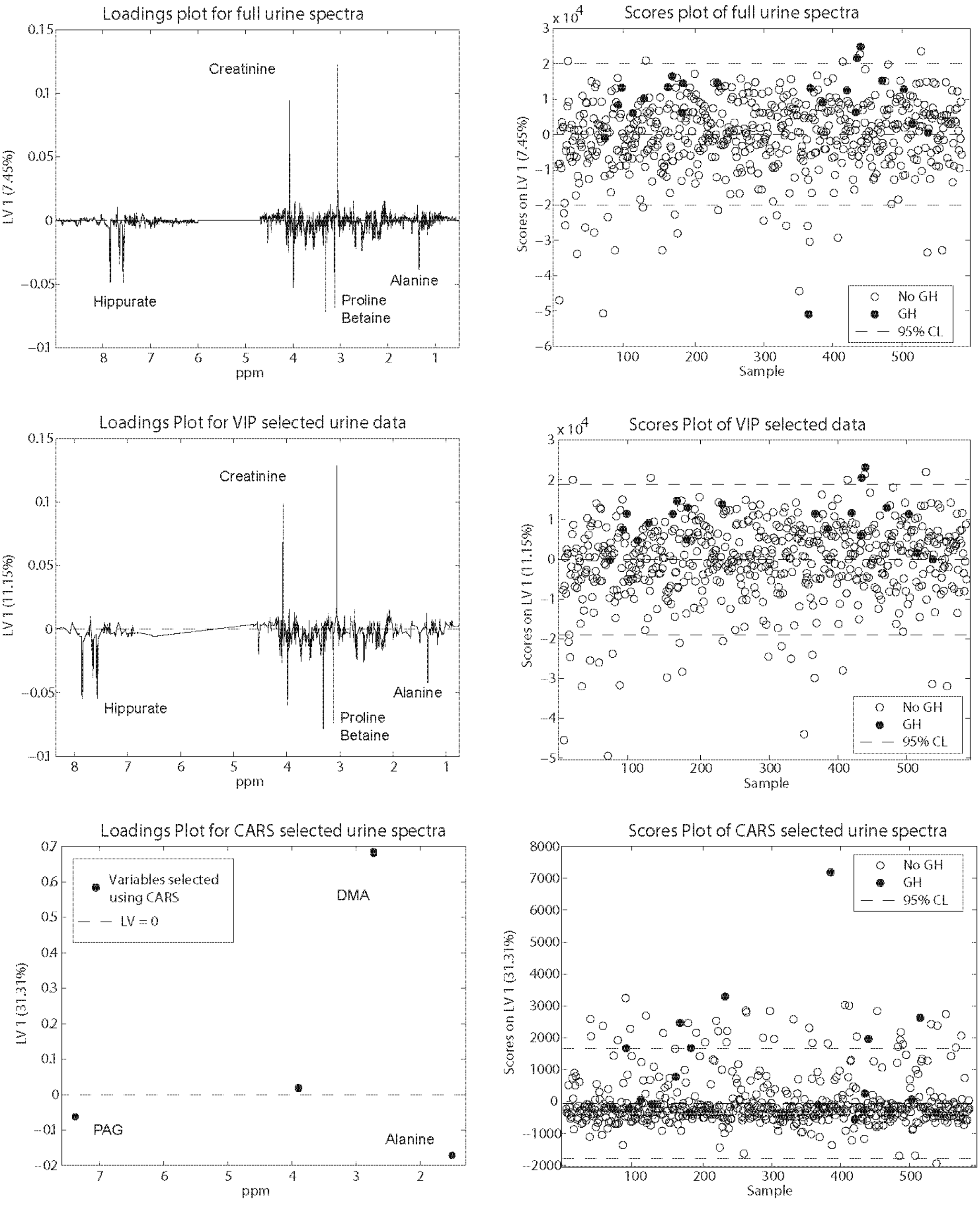

Figure S3. Scores and loadings for models predicting gestational hypertension with urine metabolomics. Abbreviations: 4-DEA, 4-deoxythreonic acid; CARS, competitive adaptive reweighted sampling; CL, confidence limit; GH, gestational hypertension; DMA, dimethylamine; LV, latent variable; ppm, parts per million; VIP, variable importance in projection.

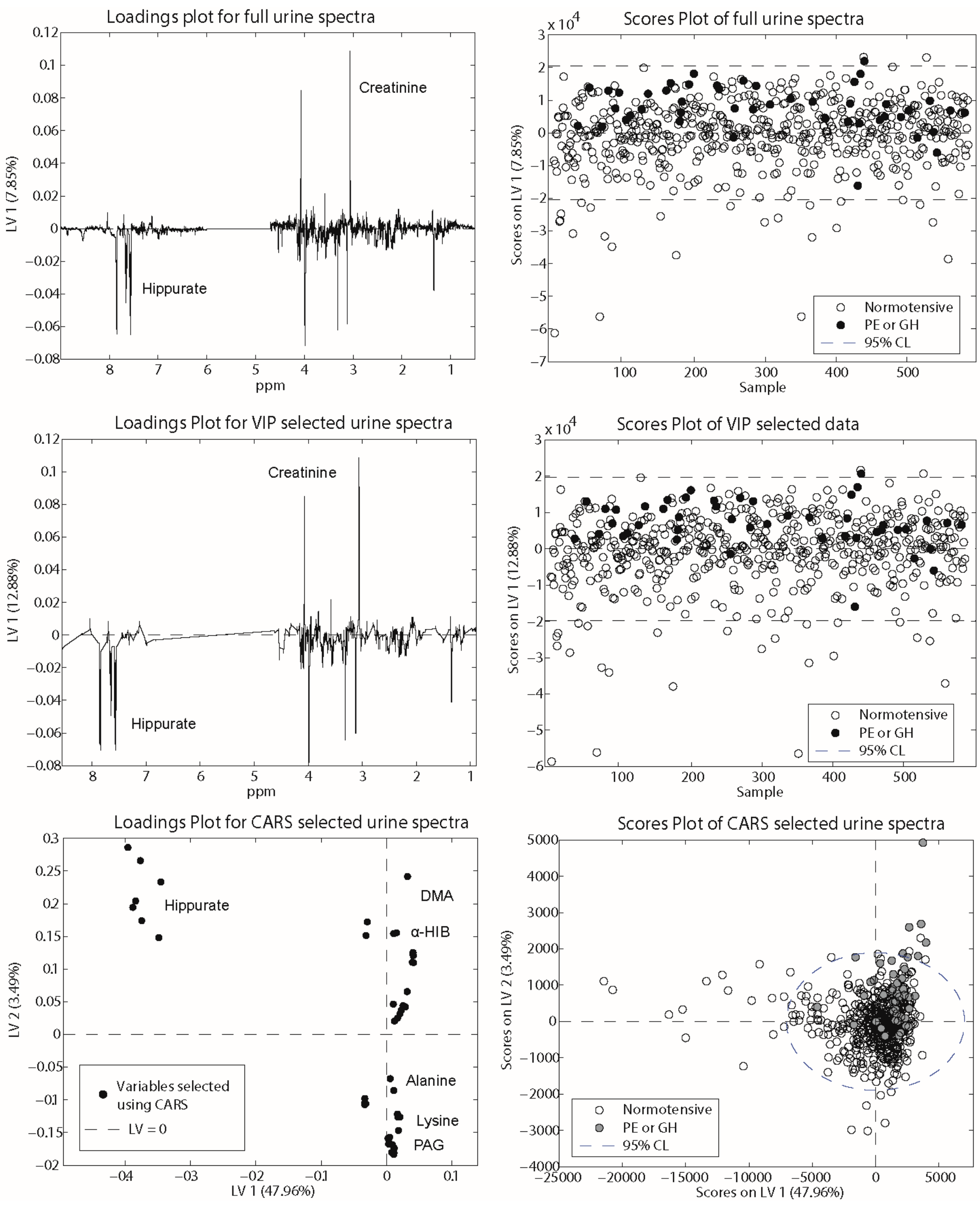

Figure S4. Scores and loadings for models predicting hypertensive disorders of pregnancy with urine metabolomics. Abbreviations: 4-DEA, 4-deoxythreonic acid; α-HIB, α-hydroxyisobutyrate; CARS, competitive adaptive reweighted sampling; CL, confidence limit; DMA, dimethylamine; GH, gestational hypertension; LV, latent variable; PAG, phenylacetylglutamine; PE, preeclampsia; ppm, parts per million; VIP, variable importance in projection.

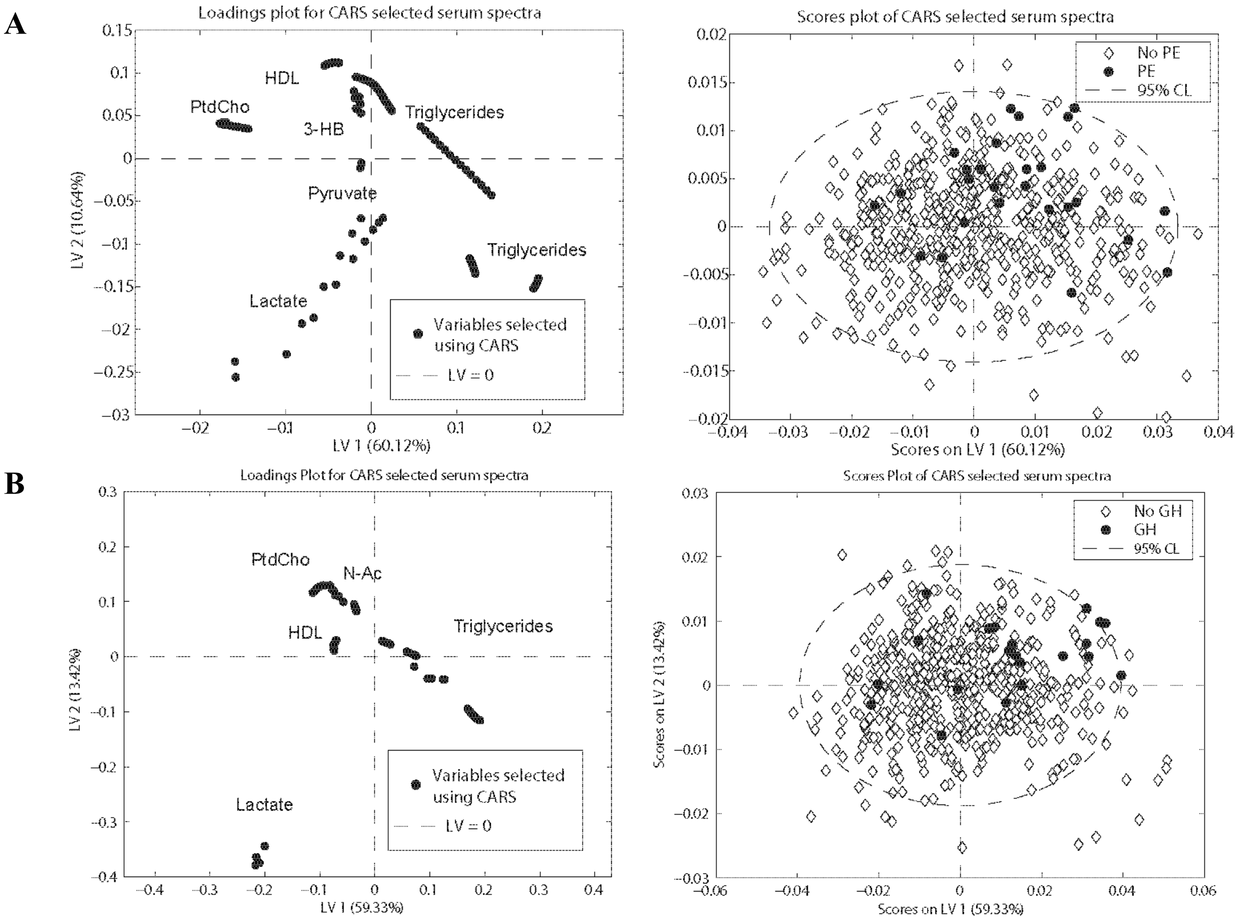

Figure S5. (**A**) Scores and loadings for models predicting preeclampsia with serum metabolomics; (**B**) Scores and loadings for models predicting gestational hypertension with serum metabolomics. Abbreviations: 3-HB, 3-hydroxybutyrate; CARS, competitive adaptive reweighted sampling; CL, confidence limit; GH, gestational hypertension; HDL, high density lipoprotein; LV, latent variable; N-Ac, N-acetyl glycoproteins; PE, preeclampsia; ppm, parts per million; PtdCho, phosphatidylcholines.

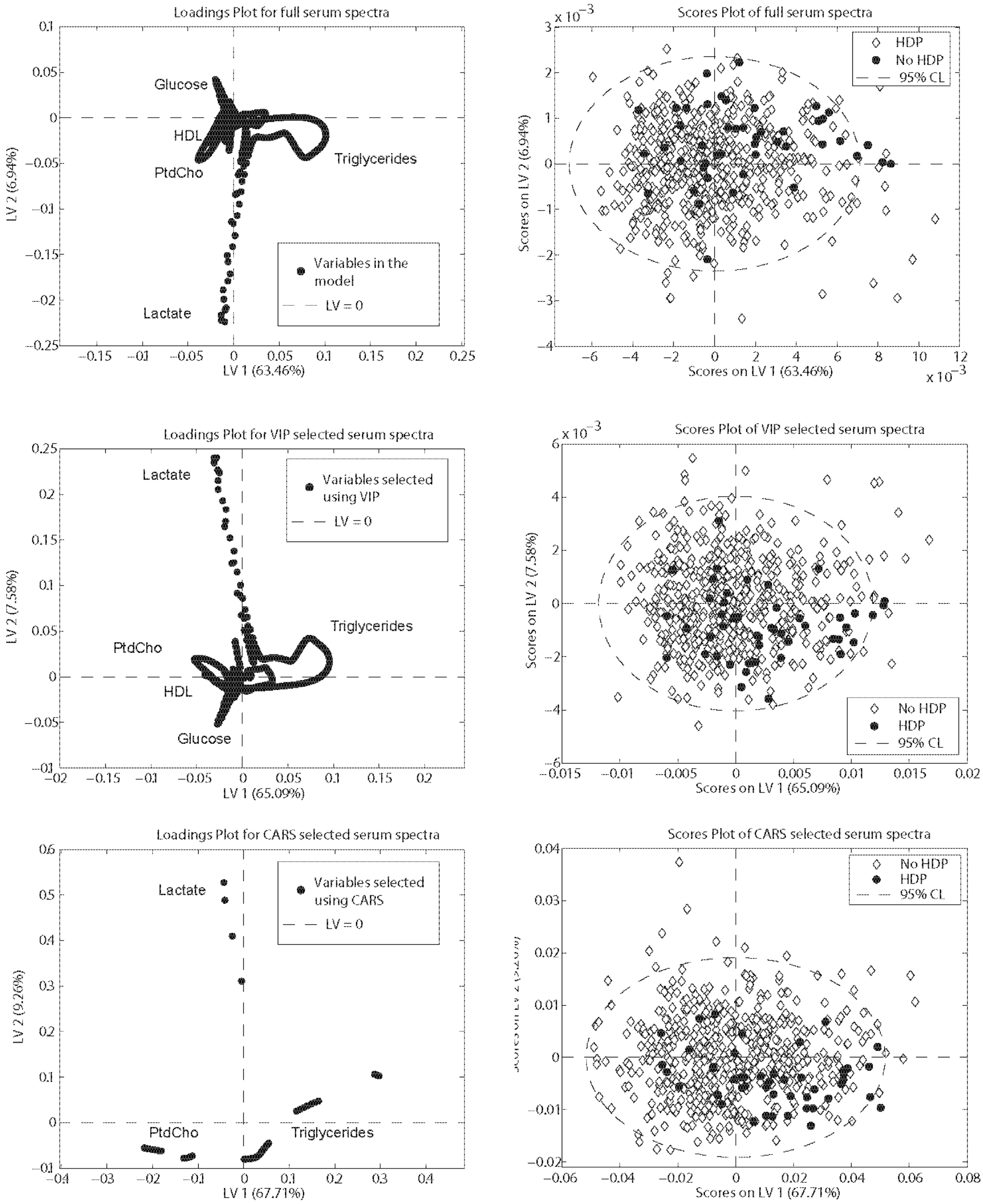

Figure S6. Scores and loadings for models predicting hypertensive disorders of pregnancy with serum metabolomics. Abbreviations: CARS, competitive adaptive reweighted sampling; CL, confidence limit; GH, gestational hypertension; HDL, high density lipoprotein; HDP; hypertensive disorders of pregnancy (PE or GH); LV, latent variable; PE, preeclampsia; ppm, parts per million; PtdCho, phosphatidylcholines.

Table S1. Metabolites Identified in ^{1}H Urine NMR spectra with aid from HSQC.

Metabolite	^{1}H NMR Shift, Multiplicity [a], (^{13}C Shift) [b]
1-Methylhistidine	3.140m (30.9), 3.730m (36.5), 3.930s (56.0), 7.210t (122.0), 8.220s (140.0), (130)
1-Methylnicotinamide	4.469s (51.4), 8.886d (146.4), 8.957d (150.0)
3-Aminoisobutyrate	1.200t (17.8), 2.037m (46.1)
3-Hydroxyisobutyrate	1.075d (16.5), 2.490m (47.6), 67.6m (186.9)
3-Hydroxyisovalerate	1.274s (31.0), 2.370s (52.3), (72.5)
3-Methylhistidine	3.270m (27.6), 3.320m (27.6), 3.770m (35.0)
4-Deoxyerythronic acid	1.11d (18.26), 4.08d (78.67), 4.1m (71.54)
4-Deoxythreonic acid	1.236d (21.2), 3.85m (79.1), 4.06m (71.7), (176.8)
4-Hydroxyphenylacetate	3.450s (46.8), 6.850d (118.2), 7.16d (133.0)
Acetate	1.93s (26.1)
Acetoacetate	2.287 (32.7), 3.343s (55.5)
Alanine	1.489d (19.1), 3.809q (53.3)
Allantoin	5.390m (65.9)
Ascorbic acid	3.770s (65.3), 4.520d (81.1), (178)
Betaine	3.270 (55.5), 3.890 (69.0)
Carnitine	2.450 (45.7), 3.202 (56.4), 3.430 (73.0)
Choline	3.230 (57.1), 3.520 (70.1)
Cis-aconitate	3.130 (46.2), 5.74 (127)
Citrate	2.550d (47.7), 2.700d (47.7)
Creatine	3.040s (39.6), 3.940 (56.4)
Creatinine	3.052s (32.7), 4.070s (59.0), (172), (191)
Dimethylamine	2.730s (37.3)
Dimethylglycine	2.933s (46.4)
Formate	8.500s (168.0)
Fumarate	6.528s
Glucose	5.210 (94.7)
Glucuronic acid	3.520 (74.5), 3.520 (78.4), 5.250d (94.7), 3.560 (74.0)
Glycine	3.580s (44.2)
Guanidoacetate	3.806 (47.9)
Hippurate	3.970d (46.6), 7.550t (131.0), 7.640t (134.0), 7.830t (129.0)
Histidine	3.220 (30.1), 3.282 (30.1), 4.016 (57.0), 7.167s (120.2), 8.060s (138.0), (133)
Hypoxanthine	8.177s (144.0), 8.200s (148.0)
Lactate	1.337d (22.4), 4.127q (71.5), (185)
Lysine	1.449 (24.1), 1.524 (24.1), 1.740 (29.5), 1.910 (33.0), 3.030 (42.3), 3.790 (57.0)
Methylamine	2.617s (27.8)
Methylmalonate	3.158 (44.3)
Myo-inositol	3.250 (77.1), 3.300 (76.9)
p-Cresol sulfate	2.346s (23.1), (133.0), (139.0)
Phenylacetylglutamine	2.27m (34.0), 3.676 (45.5), 4.010 (57.3), 7.352t (131.7), 7.362t (130.0), 7.428t (131.6)
Proline betaine	3.110s (48.6), 3.310s (54.8), 2.29m (69), 2.17m (79), 2.51m (28.1)
Propylene Glycol	1.150d (20.4), 3.470d (69.1), 3.550d, 3.900m (70.6)

Table S1. *Cont.*

Metabolite	^{1}H NMR Shift, Multiplicity [a], (^{13}C Shift) [b]
Pyruvate	2.382s (29.3)
Scyllo-inositol	3.366s (76.6)
Succinate	2.363s (36.8)
Tartaric acid	4.350s (76.7)
Taurine	3.280 (50.3), 3.440 (38.4)
Threonine	1.337d (22.4), 4.273q (68.8), (182.4)
Trigonelline	8.835t (148.0), 9.121s (148.4)
Trimethylamine-N-Oxide	3.280 (62.5)
Tyrosine	6.900 (118.0), 7.190 (133.7)
Valeric acid	0.880m (15.9), 0.930m (15.9), 1.560 (28.0), 2.200 (40.0)
Valine	0.995d (19.5), 1.050d (20.9), 2.276 (34.4), (63.3)
α-Hydroxyisobutyric acid	1.365s (29.5), (76.5), (187.1)
α-Ketoglutarate	3.01 (57.3)

[a] Multiplicities annotated as s, singlet; d, doublet; t, triplet; q; quartet; m, multiplet; [b] ^{13}C chemical shifts directly coupled to the ^{1}H in Heteronuclear Single Quantum Coherence spectra.

Table S2. Metabolites Identified in Serum NMR Spectra.

Metabolite	^{1}H Shifts [a] and Multiplicities
3-Hydroxybutyrate	1.219d
Acetate	1.935s
Acetoacetate	2.300s
Acetone	2.248s
Alanine	1.499d
Asparagine	2.871d, 2.951d
Citrate	2.554d, 2.701d
Creatine	3.059s, 3.948s
Creatinine	3.062s, 4.072s
Formate	8.478s
Glucose	3.269t, 3.403–3.576m, 3.716–3.939m
Glutamine	2.152m, 2.475m, 3.772m
Glycerol	3.677m
Glycine	3.58s
Histidine	7.808s, 7.081s, 3.14m
Isoleucine	1.028d
Lactate	1.347d, 4.131q
Leucine	0.983d, 0.963d, 1.734m
Lysine	1.916m
Methanol	3.38s
Methionine	2.662t
N-Acetylated groups	2.059s
Phenylalanine	7.449t, 7.393t, 7.352d
Proline	2.37m, 3.354m
Propylene Glycol	1.139d
PtdCho	3.238b
Pyruvate	2.39s
Threonine	3.593d, 1.347d, 4.271q
Tyrosine	7.217d, 6.920d
Valine	1.06d, 1.009d, 3.63d

[a] Multiplicities annotated as s, singlet; d, doublet; t, triplet; q; quartet; m, multiplet; b, broad signal.

Table S3. Additional prediction model parameters for urine spectra.

Pregnancy Outcome	Variable Selection Method	No. Variables	No. Latent Variables in Prediction Model	*p*-Value
PE (*n* = 26)	None	27,084	1	<0.01
	VIP ≥ 1	3747	1	<0.01
	CARS	54	1	<0.01
GH (*n* = 20)	None	27,084	1	<0.01
	VIP ≥ 1	4653	1	0.01
	CARS	8	1	0.04
PE + GH (*n* = 46)	None	27,084	1	<0.01
	VIP ≥ 1	3789	1	<0.01
	CARS	45	4	<0.01

Abbreviations: CARS, Competitive adaptive reweighted sampling; GH, Gestational Hypertension; PE, preeclampsia; VIP, Variable importance in projection. The *p*-values are from 100 permutation tests.

Table S4. Additional prediction model parameters for serum spectra.

Pregnancy Outcome	Variable Selection Method	No. Variables	No. Latent Variables in Prediction Model	*p*-Value
PE (*n* = 26)	None	13,420	4	>0.05
	VIP ≥ 1	1081	7	>0.05
	CARS	105	3	0.05
GH (*n* = 20)	None	13,420	1	>0.05
	VIP ≥ 1	1259	7	>0.05
	CARS	87	4	0.02
PE + GH (*n* = 46)	None	13,420	4	0.01
	VIP ≥ 1	1328	4	<0.00
	CARS	51	6	0.02

Abbreviations: CARS, Competitive adaptive reweighted sampling; GH, Gestational Hypertension; PE, preeclampsia; VIP, Variable importance in projection. The *p*-values are from 100 permutation tests.

Table S5. NMR spectroscopy parameters.

Biofluid	Spectrum Shorthand	Pulse Sequence	Temp (K)	FID Size	Scans	Spectral Width (ppm)
Urine	NOESY	noesygppr1d	300	65,536	32	20.5682
	JRES	jresgpprqf	300	8192 × 40	2	16.6602 × 0.1302
	HSQC	hsqcedetgpsisp.2	300	2048 × 256	32	16.0194 × 165.6500
	COSY	cosygpprqf	300	4096 × 512	8	16.0194 × 16.0194
	HSQC-TOCSY	hsqcdietgpsisp.2	300	2048 × 256	32	12.0146 × 165.6580
	HMBC	hmbcetgpl3ndpr	300	4096 × 256	128	16.0194 × 209.9990
Serum	NOESY	noesygppr1d	310	98,304	32	29.8927
	CPMG	cpmgpr1d	310	65,536	64	20.0243
	JRES	jresgpprqf	310	8192 × 40	1	16.6602 × 0.1302

Abbreviations: COSY, Correlated spectroscopy; CPMG, Carr-Purcell-Meiboom-Gill pulse sequence; HMBC, Heteronuclear multiple bond correlation spectroscopy; HSQC, Heteronuclear single quantum coherence spectroscopy; JRES, J-resolved spectroscopy; NOESY, Nuclear overhauser effect spectroscopy; TOCSY, Total correlation spectroscopy.
